# Supplementary material for: TRAIP resolves DNA replication-transcription conflicts during the S-phase of unperturbed cells
Source: Nat Commun. 2023 Aug 21;14:5071. doi: 10.1038/s41467-023-40695-y (PMC10442450; doi:10.1038/s41467-023-40695-y)
Supplement: Supplementary file 1 — Supplementary Information [file 41467_2023_40695_MOESM1_ESM.pdf]

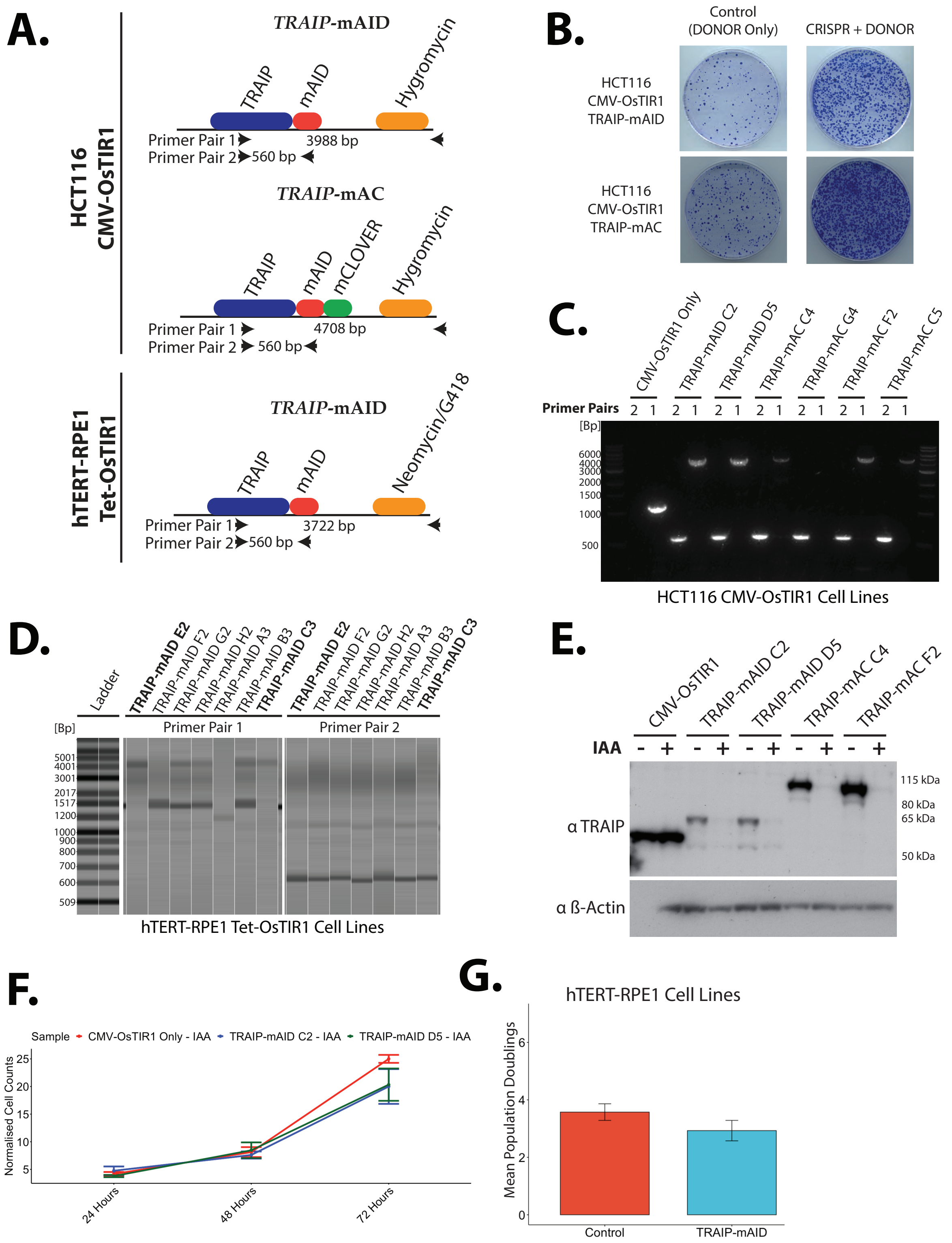

Supplementary Figure 1. Creation and validation of TRAIP degrons in HCT116 and RPE1 cells. (A) Design of TRAIP tagged with auxin inducible degron (mAID) or mClover tagged degron (mAC). Primer pairs used for verification of correct genomic incorporation is presented below the diagrams. **(B)** Examples of cell colonies obtained after guide RNAs transfection stained with crystal violet. **(C)** Validation of biallelic tagging of TRAIP in different HCT116 clones using primer pairs indicated in (A) (n=2) **(D)** Validation of biallelic tagging of TRAIP in different RPE1 clones using primer pairs indicated in (A). **(E)** Validation of biallelic tagging and tagged TRAIP degradation in HCT116 cells by western blotting with TRAIP antibodies (n=3) **(F)** Growth curves of control (CMV-OsTIR1) and two clones of HCT116 TRAIP-mAID cells (n = 3). Hypothesis testing was carried out using one-way ANOVA for each individual timepoint (24 h: Df=2, F=0.892, p=0.458. 48 h: Df=2, F=0.18, p=0.839. 72 h: Df=2, F=1.236, p=0.355). **(G)** Mean number of population doublings over 72 h growth in hTERT-RPE1 TRAIP-mAID cells or hTERT-RPE1 Tet-OsTIR1 parental cell lines. n = 2. Source data are provided as a Source Data file.

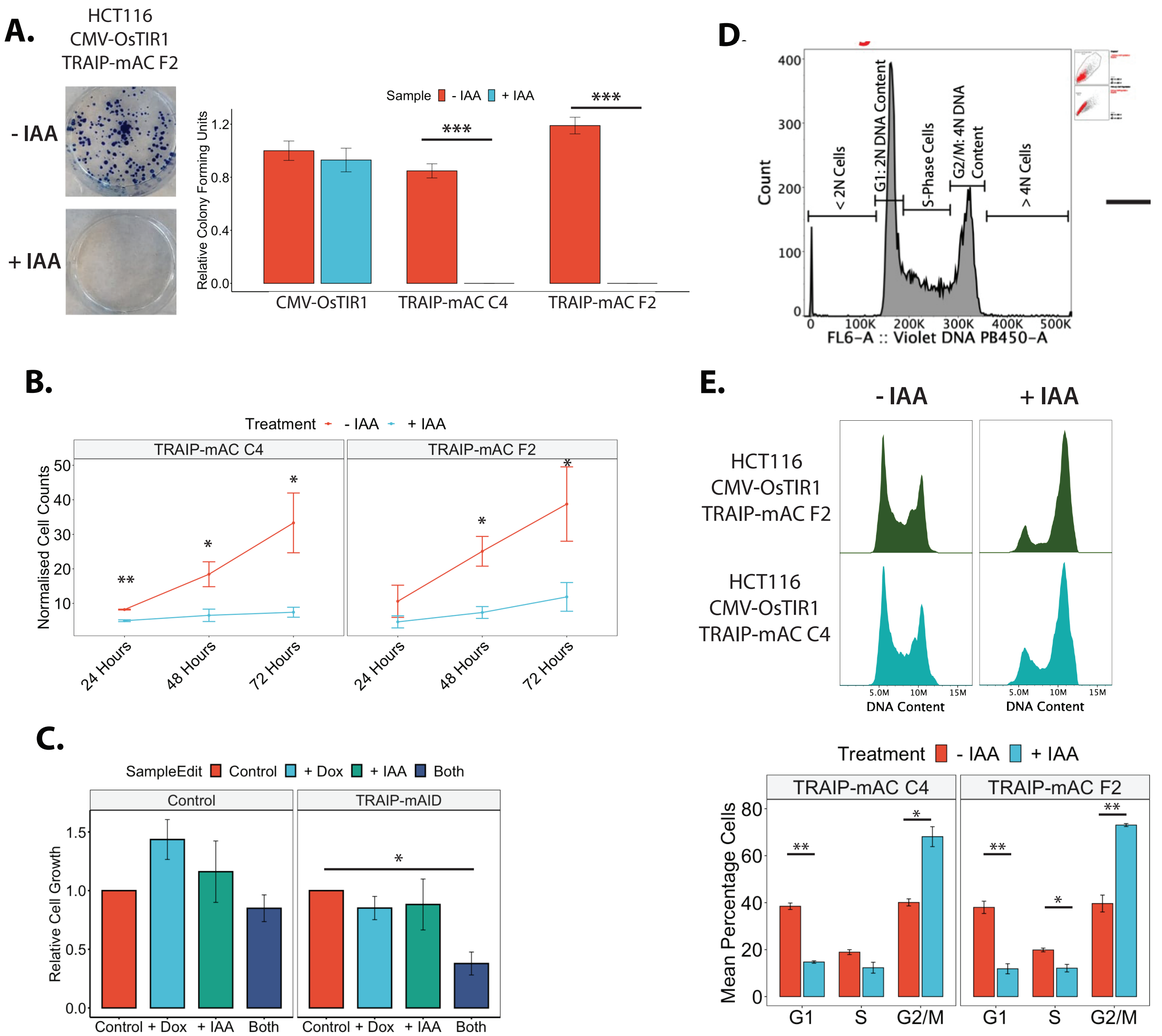

Supplementary Figure 2. Phenotypes of inhibited proliferation and cell cycle arrest upon TRAIP degradation are reproduced in HCT116 TRAIP-mAC and RPE1 cell lines. (A) Example of colony assay after auxin treatment. Cells were fixed and stained with methylene blue (left). Quantification of colony forming assay in two independent TRAIP-mAC clones n=3 (right). Statistical analyses was carried out using T.tests. Significant differences were identified between TRAIP-mAC C4 (p=0.000161) and TRAIP-mAID F2 (p=0.000159). **(B)** Growth curves of TRAIP-mAC cells upon 24, 48 and 72 h of auxin treatment. Cells were counted at every timepoint and normalised to the seeding densities n=3. Statistical significance was calculated using T.tests for TRAIP-mAC C4 (24 hours: p=0.002158; 48 hours: p=0.03; 72 hours: p=0.04613) and TRAIP-mAC F2 (48 hours: p=0.0399; 72 hours: p=0.03326). **(C)** hTERT-RPE1 control (Tet-osTIR1) and TRAIP-mAID cells were optionally treated for 72 h with doxycycline and/or auxin as indicated. Statistical analyses - T.tests p = 0.02392. Significant differences were detected in hTERT-RPE1 TRAIP-mAID untreated control vs Both treatments only. **(D)** Example of FACS gating strategy used for cell cycle analysis. Total DNA content, with gates applied to identify potential apoptotic cells (< 2N DNA content), G1 cells (2N DNA Content), G2/M Cells (4N DNA content), S-phase, and any cells with re-replication (> 4N DNA content). The small boxes to the top right depict the gating strategy used for isolating primary cell populations and removing doublets **(E)** TRAIPmAC clones arrest at G2/M stage of the cell cycle. Examples of FACS cell cycle profiles of two TRAIP-mAC clones upon 24 h of optional auxin treatment (above). Quantification of cell cycle stages in TRAIP-mAC clones after 24 h auxin treatment n=3 (below). Statistical analysis (t.tests) - G1: TRAIP-mAID C4 (p=0.00122), and TRAIP-mAID F2 (p=0.00175); G2/M - TRAIP-mAC C4 (p=0.0153), TRAIP-mAC F2 (p=0.00978). Source data are provided as a Source Data file.

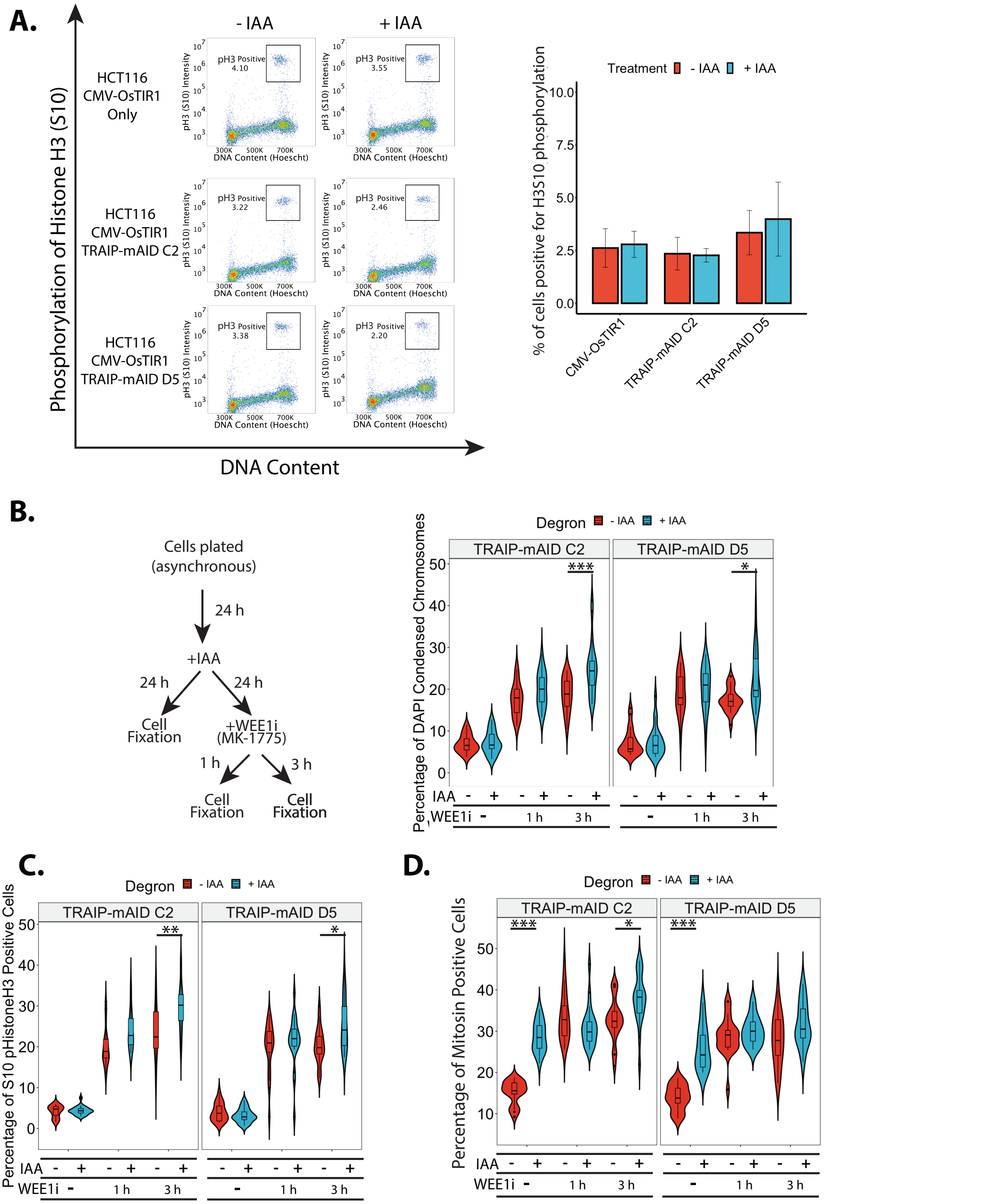

Supplementary Figure 3. TRAIP depleted cells accumulate in G2 stage of the cell cycle. (A) HCT116 degron cell lines were treated with auxin for 24 h and stained for S10 phosphorylation on histone H3 (pH3). Example FACS plots with pH3 staining against DNA content. Mitotic cells are selected in the black box (left). Quantification of the percentage of cells in mitosis (pH3 positive) upon 24 h of TRAIP degradation n=3 (right). Statistical analysis revealed no differences in the data. **(B)** Schematic of the experiment (left) and quantification of the percentage of cells found to be in mitosis after 24 h of auxin treatmentby quantification of cells with condensed chromosomes after DAPI staining and fluorecent microscopy (mitotic index) (right). Significance (ANOVA and pairwise testing) is summarised on the plot: TRAIP-mAID C2 ($p = < 0.001$) and TRAIP-mAID D5 ($p = 0.0162$). **(C)** Quantification of H3S10 phosphorylation by fluorescent microscopy in TRAIP degron cell upon 24 h of auxin treatment and optional treatment with WEE1 inhibitor MK-1775. Significance (ANOVA and pairwise testing) is summarised on the plot: TRAIP-mAID C2: $p = 0.00260$; TRAIP-mAID D5: $p = 0.0170$. **(D)** Quantification of mitosin positive cells upon treatment as in (C). Significance (ANOVA and pairwise comparison) is summarised on the plot: TRAIP-mAID C2 Untreated ($p = < 0.001$), TRAIP-mAID C2 + Wee1i 3 hours ($p = 0.0294$), and TRAIP-mAID D5 untreated ($p = < 0.001$). Source data are provided as a Source Data file.

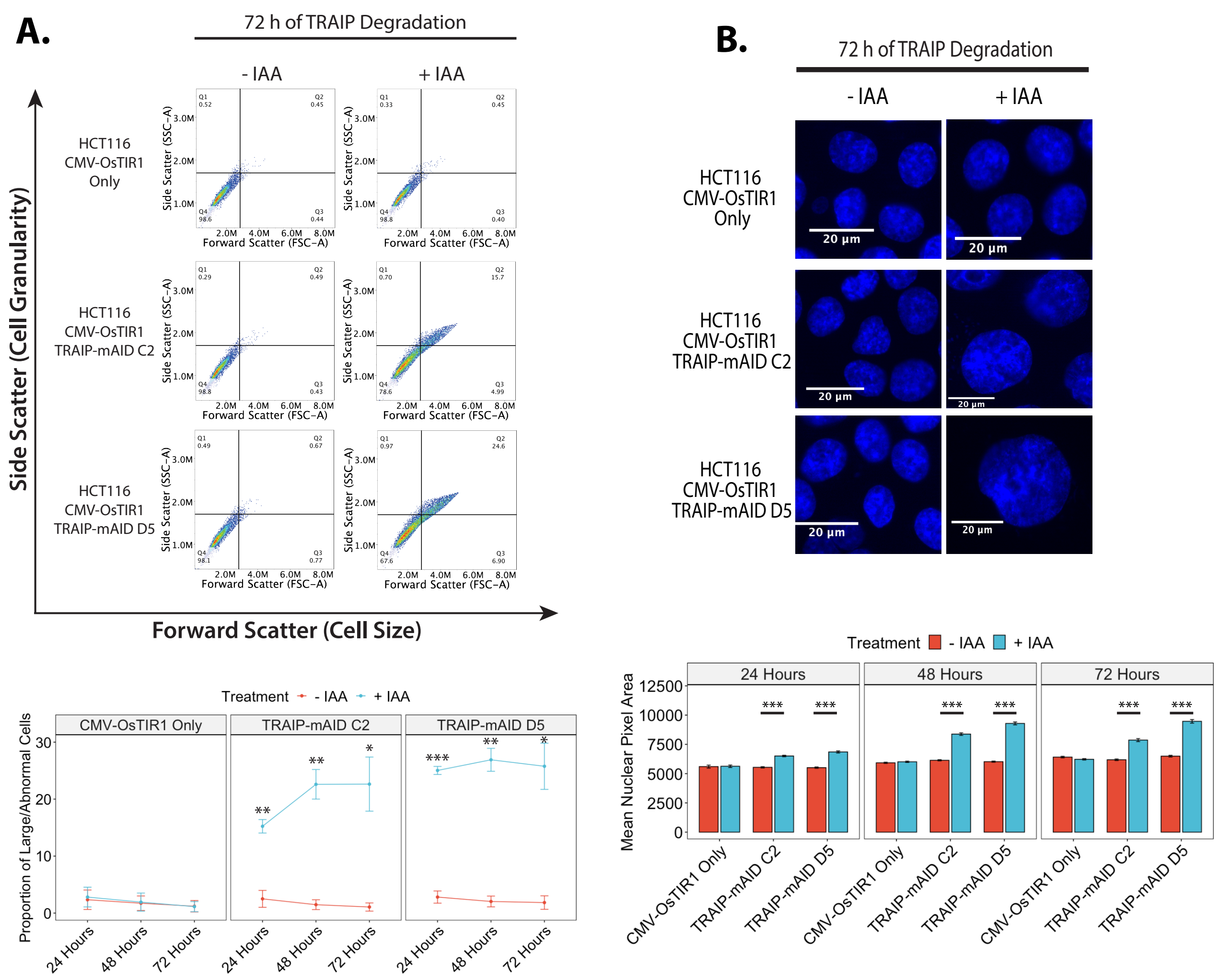

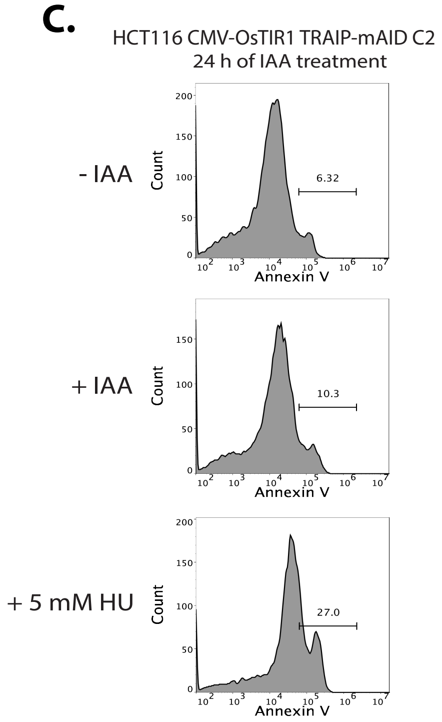

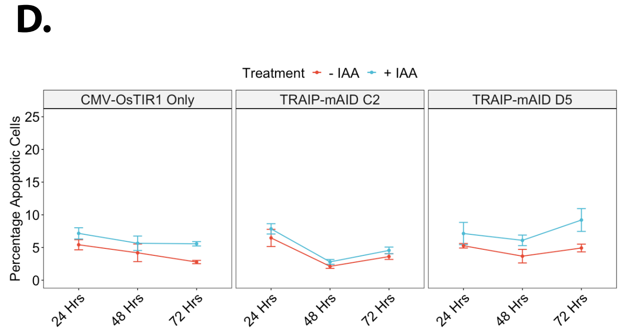

Supplementary Figure 4. Analysis of consequences of TRAIP depletion. (A) The size of TRAIP degron cells was analysed by flow cytometry after auxin addition. The example FACS plots after 72 h of IAA treatment (top) and quantification over 24, 46 and 72h TRAIP degradation n=3 (bottom) is presented. **(B)** The size of nuclei was analysed by DAPI staining and fluorescence microscopy. Example pictures after 72 h of IAA treatment (top) and quantification over 24, 46 and 72h TRAIP degradation n=3 (bottom) is presented. **(C)** Annexin V staining assay validation. TRAIP-mAID cell lines were treated optionally with auxin for 24 h or HU for 24 h. Annexin V positive cells were detected by FACS. Example FACS profiles presented.
(D) Quantification of percentage of TRAIP degron cells positive for Annexin V after 24, 48 or 72 h of auxin treatment. Source data are provided as a Source Data file.

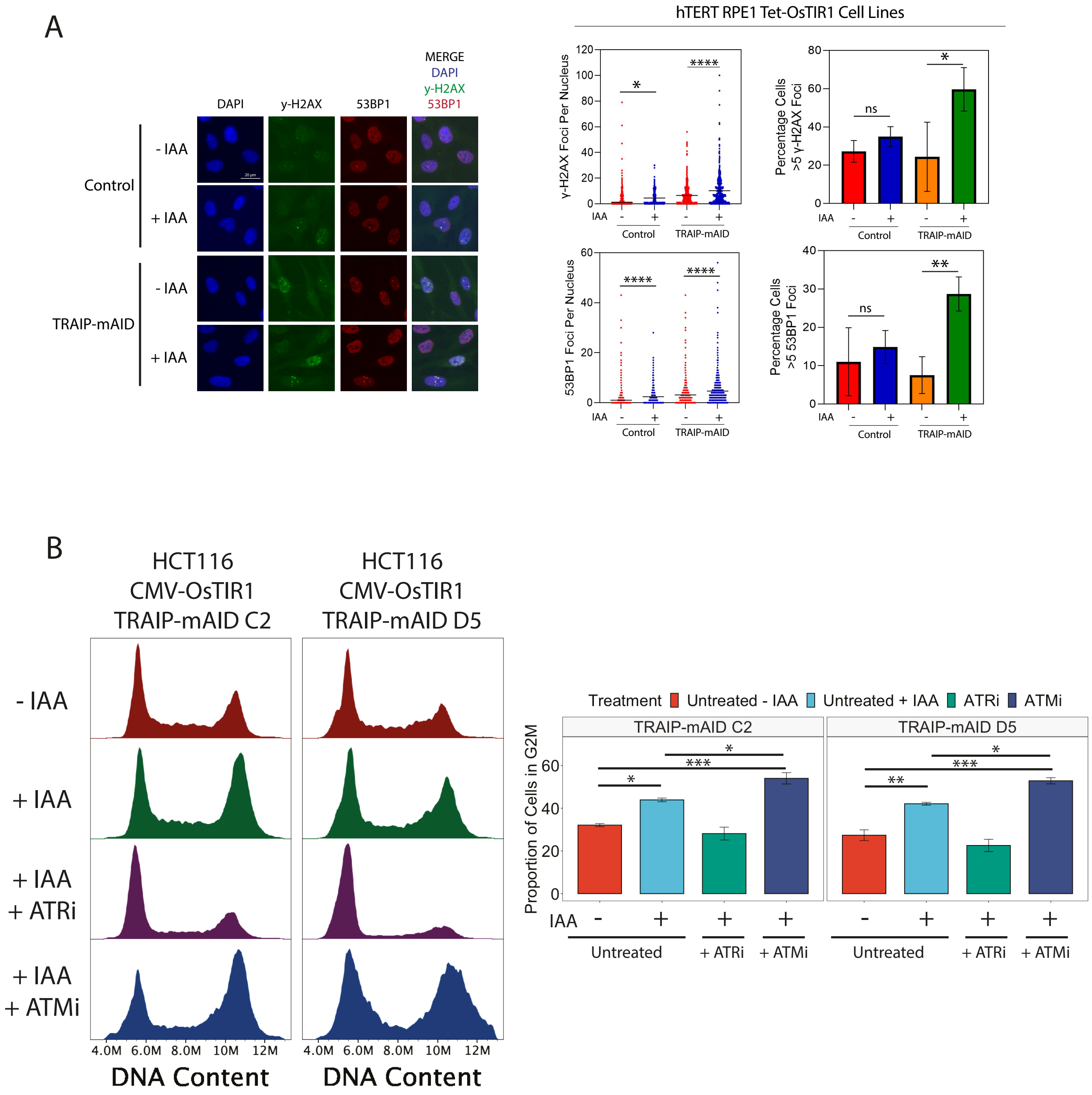

Supplementary Figure 5. (A) Accumulation of DNA damage in RPE1 TRAIP-mAID cells upon auxin treatment. Example photos (left), total number of foci detected per nucleus over n=3 (middle). Mann -Whitney test performed. γ-H2AX foci/nucleus control -IAA vs +IAA; p=0.0154, TRAIP-mAID -IAA vs +IAA; p<0.0001. 53BP1 foci/nucleus control -IAA vs +IAA; <0.0001, TRAIP-mAID -IAA vs +IAA; <0.0001. T.test performed for cells with >5 γ-H2AX foci TRAIP-mAID -IAA vs +IAA; p=0.018. >5 γ-H2AX foci TRAIP-mAID -IAA vs +IAA; p=0.006.

(B) G2 arrest in TRAIP depleted cells is dependent on functioning ATR checkpoint pathway. TRAIP degron clones were treated for 24 h with auxin and optionally with ATRi or ATMi. Cell cycle profile of cells was analysed by FACS. Example of FACS plots (left) and quantification of cells in G2/M stage of the cell cycle n=3 (right).

Source data are provided as a Source Data file.

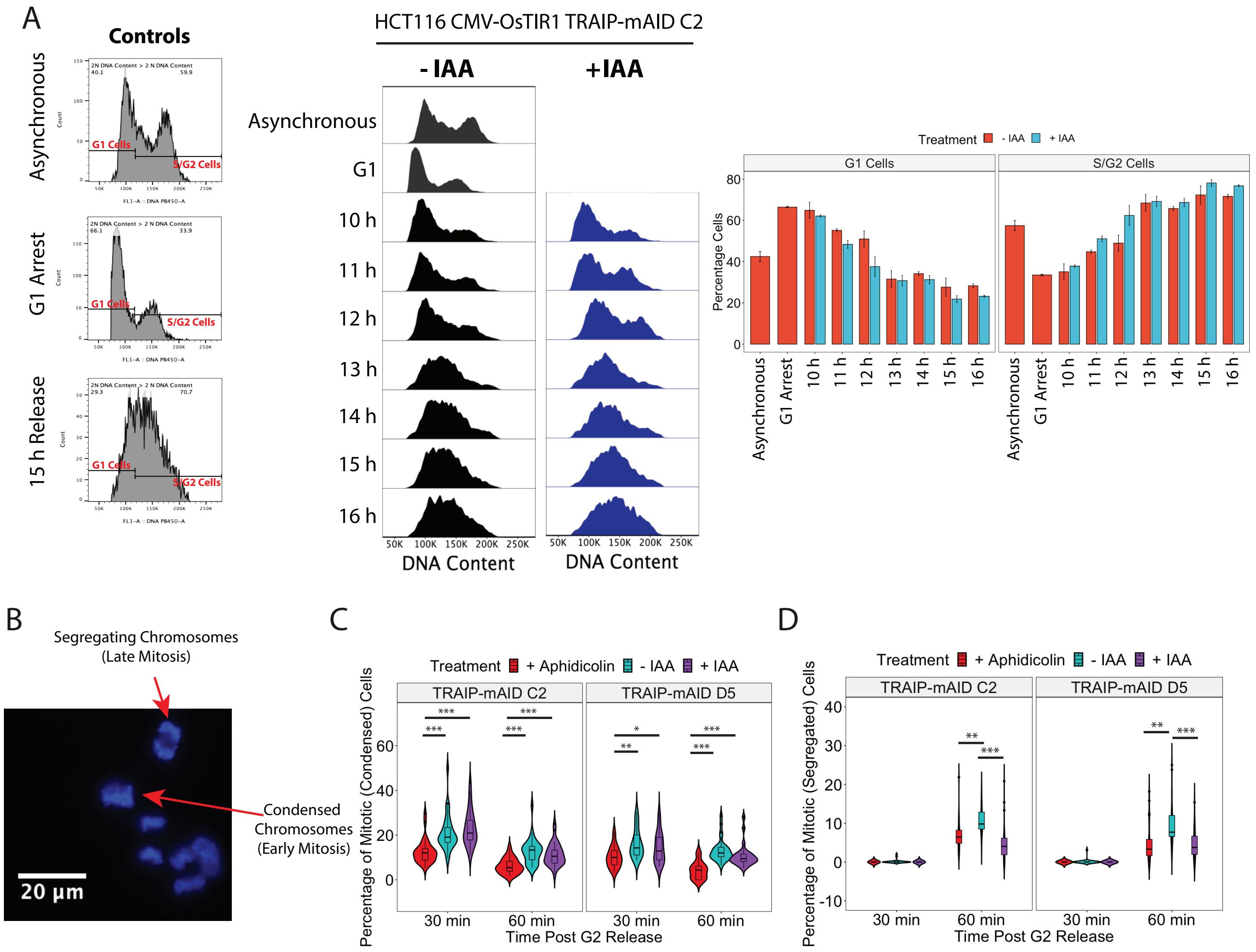

Supplementary Figure 6. TRAIP regulates mitotic progression. (A) TRAIP degradation at the end of G1 does not affect progression into S-phase. HCT116 degron cells were arrested in G1 with lovastatin, TRAIP degraded and cells released from G1 arrest. Cell cycle progression at indicated times was assesed by DNA content and flow cytometry. Left: Control arrest samples and example quantification gates indicating those cells in G1 or in S/G2. Middle: Example cell cycle profiles -/+ IAA throughout the arrest. No visual differences could be observed between treatments. Right: Quantification of G1 or S/G2 cells by the gates depicted in controls. Statistical singnificant testing was carried out using T.tests, but no differences could be found. n = 2. **(B-D)** TRAIP regulates mitotic progression. HCT116 degron cells were arrested in G2 with RO-3306. TRAIP was degraded, cells released into mitosis and progression through mitosis observed at 30 and 60 min post release. As a control cells were treated with aphidicolin during RO-3306 treatment to slow down progression through the cell cycle. **(B)** example of detection of cells with condensed and segregating chromosomes. **(C)** Quantification of percentage of cells with condensed chromosomes n = 3. Shown are aphidicolin controls (red), - IAA (blue), and + IAA (purple); depicted as violin plots showing the median, interquartile range, and overall data distribution. Statistical significance were determined using one-way ANOVA and pairwise post-hoc testing. Significance is indicated on the plot. Increased proportions of cells were shown in -IAA/+IAA samples compared to controls at all timepoints ($p < 0.001$ for all comparisons shown). Conversely, no differences were observed between -IAA and + IAA samples. **(D)** Quantification of percentage cells with segregating chromosomes. Data depicted as in (C). Statistical analysis testing was carried out using one-way ANOVA and pairwise post-hoc testing. No differences were detected at 30 minutes post release. For 60 minute timepoints, significance is indicated on the plot: TRAIP-mAID C2 60 mins + aphidicolin control vs – IAA: $p = 0.00117$, - IAA vs + IAA: $p < 0.001$; TRAIP-mAID D5 60 mins + aphidicolin control vs – IAA: $p = 0.00209$; - IAA vs + IAA: $p < 0.001$. Source data are provided as a Source Data file.

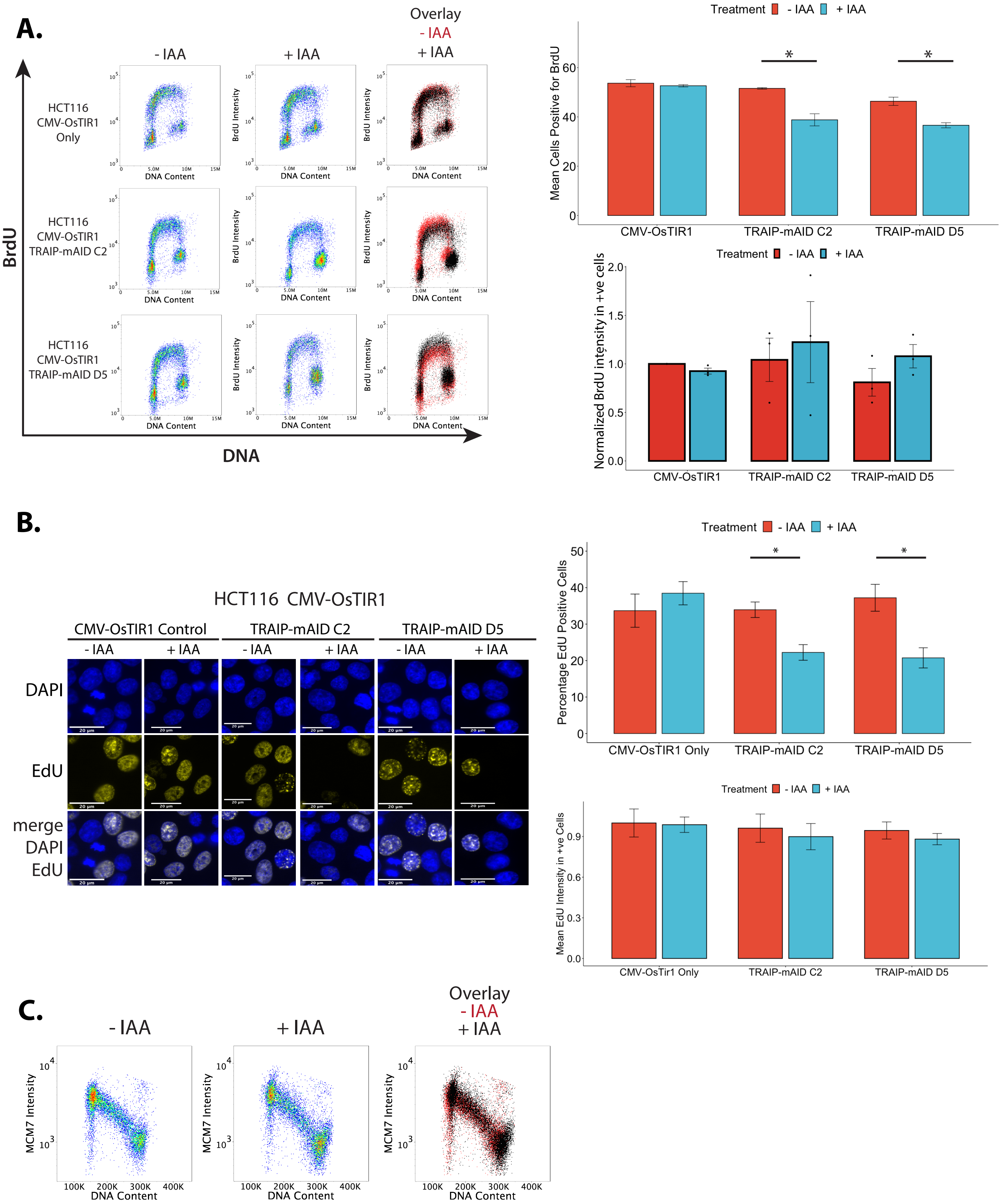

Supplementary Figure 7. TRAIP degradation does not affect global DNA replication. (A) Cells optionally treated with IAA for 24 h were pulsed with BrdU for 1 h and BrdU incorporation into DNA analysed by FACS. Example FACS plots are presented (left) and quantification of number of cells incorporating BrdU and the level of BrdU incorporation normalised to control in replicating cells over n=3 (right), mean and SEM. Statistical analysis was carried out using t. tests, and any significant differences discovered is summarised on the plot (TRAIP-mAID C2: p = 0.0331; TRAIP-mAID D5: p = 0.0126). **(B)** Cells optionally treated with IAA for 24 h were pulsed with EdU for 1 h and EdU incorporation into DNA detected by immunofluorescence. Example microscopy images are presented (left) and quantification of number of cells incorporating EdU and level of EdU signal in replicating cells over n=3 (right). Statistical analyses was carried out on the data using t. tests and significant differences are summarised on the plots (TRAIP-mAID C2: p = 0.0178; TRAIP-mAID D5: p = 0.0264). **(C)** Cells were optionally treated with IAA for 24 h, nuclei extracted and the level of chromatin bound MCM7 analysed by FACS. Source data are provided as a Source Data file.

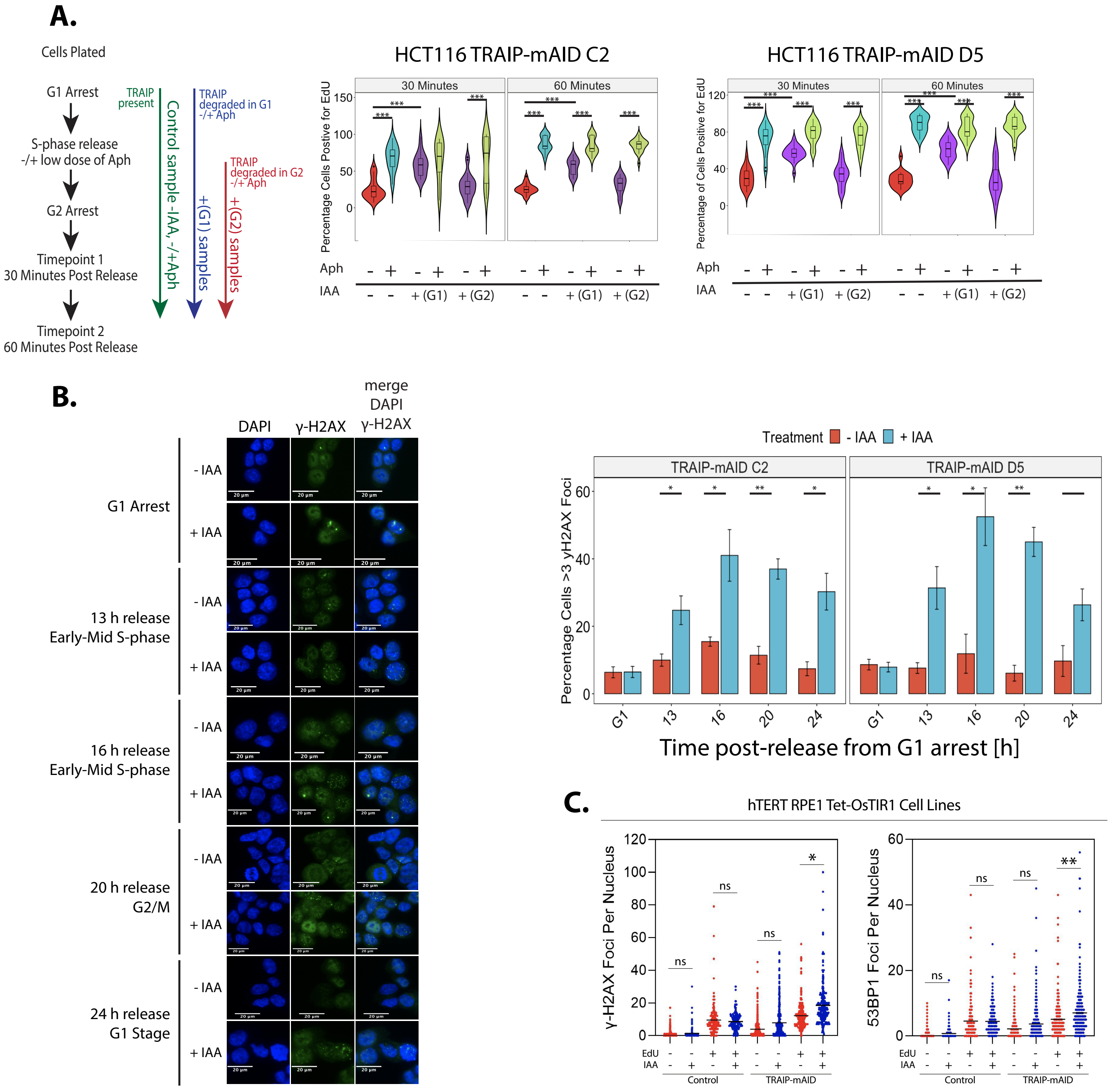

Supplementary Figure 8. DNA replication defect and DNA damage observed in cells after TRAIP depletion. (A) TRAIP Degradation Prolongs DNA Replication into G2/M. HCT116 degron cells were synchronised and treated as depicted in the schamatic (left). Quantification of the total proportion of cells positive for EdU incorporation for experiments using TRAIP-mAID C2 and TRAIP-mAID D5 (n = 3). Any differences between groups were determined using one-way ANOVA testing and post-hoc pairwise comparisons, indicated on the plots ($p > 0.001$ for all comparisons shown). (**B**) TRAIP was degraded just before S-phase as in (A). At indicated time points the DNA damage foci (γH2AX and 53BP1 foci) were analysed by immunofluorescence. Example images are presented (left) and quantification of percentage of cells with over 3 γH2AX foci over n=3 experiments (right). Significant differences (t.tests) are summarised on the graph for TRAIP-mAID C2 (13 Hrs post release: p = 0.028; 16 Hrs post release: p = 0.037; 20 Hrs post release, p = 0.0016; 24 Hrs post release, p = 0.019) and TRAIP-mAID D5 (13 Hrs post release: p = 0.028; 16 Hrs post release, p = 0.010; 20 Hrs post release, p = 0.0019; 20 Hrs post release, p = 0.032). **(C)** Accumulation of DNA damage in RPE1 TRAIP-mAID cells upon auxin treatment in EdU-negatuve vs EdU-positive cells. Students T-tests performed: γ-H2AX TRAIP-mAID -IAA vs +IAA (EdU pos); p=0.045. 53BP1 TRAIP-mAID -IAA vs +IAA (EdU pos); p=0.002. Source data are provided as a Source Data file.

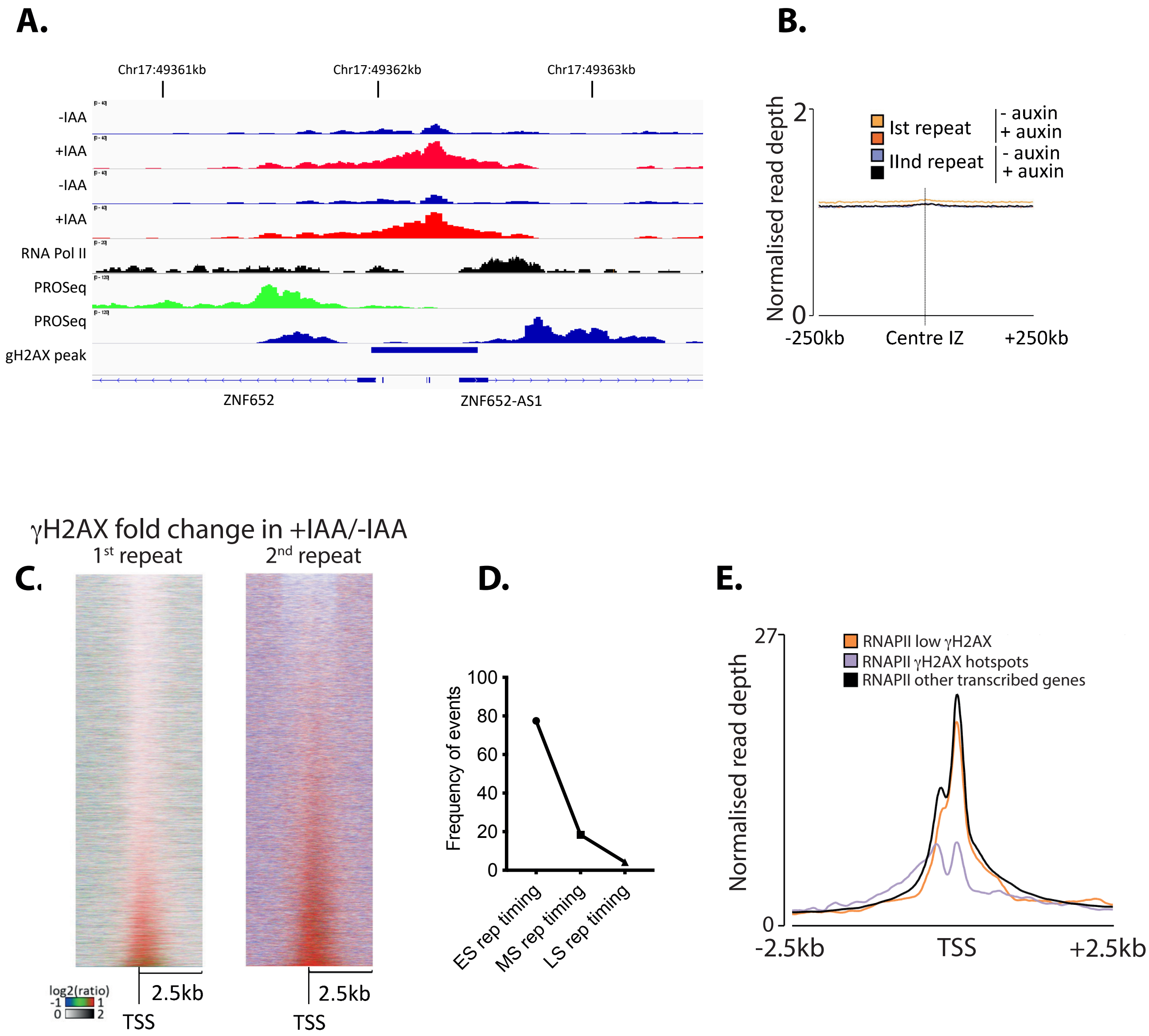

Supplementary Figure 9. The increase of γH2AX upon TRAIP degradation does not correlate with replication features but with transcription start sites. (A) An example of mapped γH2AX peak. Similar to the example in Figure 4A. **(B)** Correlation of γH2AX signal with replication initiation and termination zones as determined by Daigaku et al. 2022. **(C)** Heatmaps of γH2AX signal centered on transcription start sites (TSS). TSS sorted by γH2AX fold change in +IAA/-IAA. Two independent repeats are presented. **(D)** Replication timing of hotspots. **(E)** Metagene profile of RNA Pol II ChIP-Seq from Erickson et al. at the TSS +/- 2.5 kb for genes with a gH2AX hotspot following TRAIP degradation, genes with low enrichment levels of gH2AX, and all the other transcribed genes, showing clear differences in the profile and distribution of RNA Pol II at the TSS region. Source data are provided as a Source Data file.

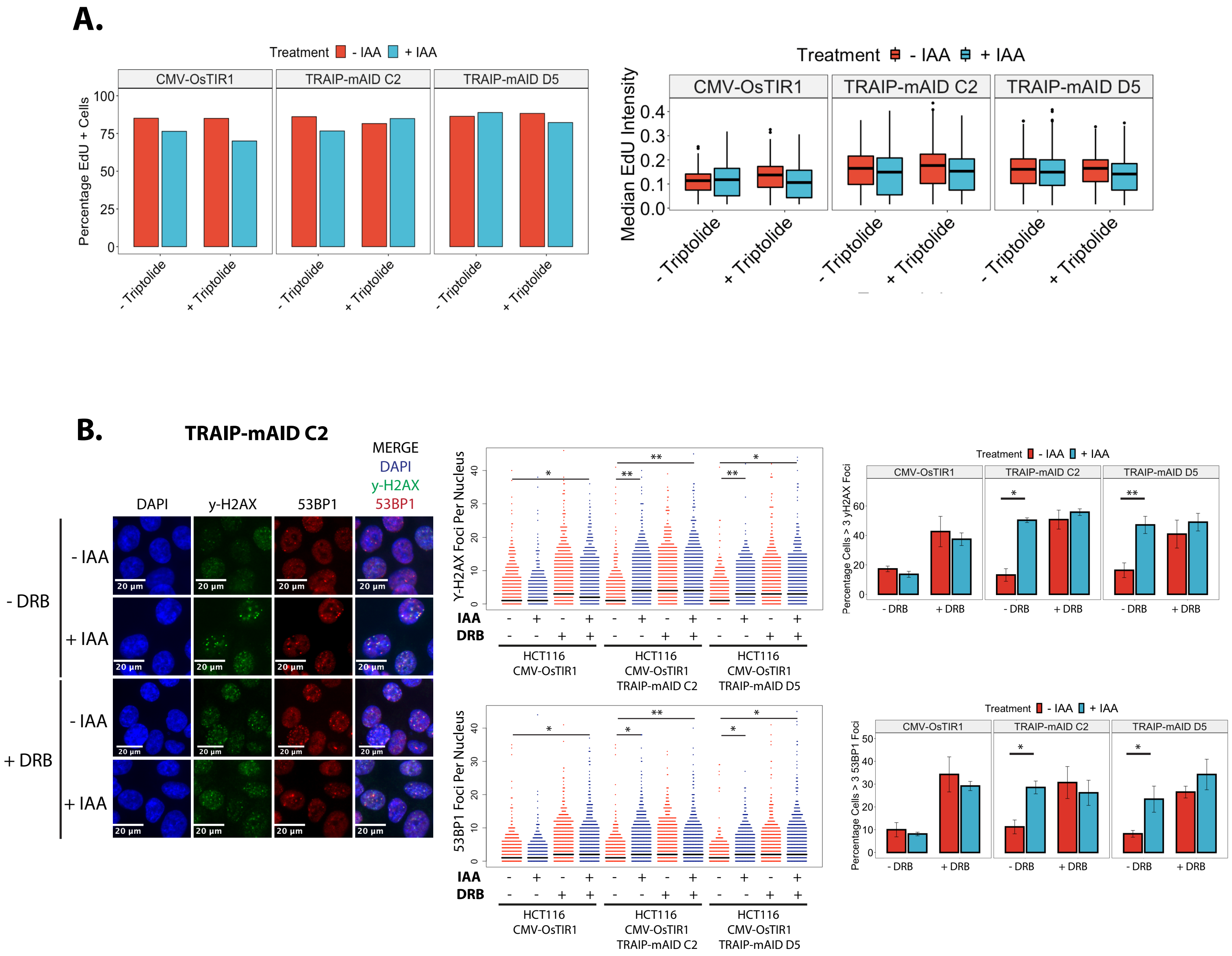

Supplementary Figure 10. (A) Short Triptolide treatment in S-phase does not inhibit DNA replication. Cells were synchronised in G1 with optional degradation of TRAIP for last 1 h of arrest and released into mitosis. Cells were then treated for 90 min with Triptolide, 3 h after release from G1 arrest. Cells were pulsed for 1 h with EdU and its incorporation was analysed using fluorescent microscopy. Shown are the overall percentage of cells positive for EdU (left), as well as median EdU intensity per cell (right). **(B) DRB treatment during S-phase is epistatic with TRAIP degradation.** Cells were synchronised in G1, where TRAIP was degraded prior to entry to S-phase. Upon S-phase entry DRB was added for 90 minutes and cells fixed, then stained for DNA damage markers. Left: Example microscopy images. Middle: Total foci counts per nucleus. Ttest for pairwaise comparisons. γH2AX: all ** < 0.009, * < 0.036; 53PB1: ** $p = 0.00965$, all * < 0.048. Right: Quantification of the proportion of nuclei containing > 3 DNA damage foci. Source data are provided as a Source Data file.

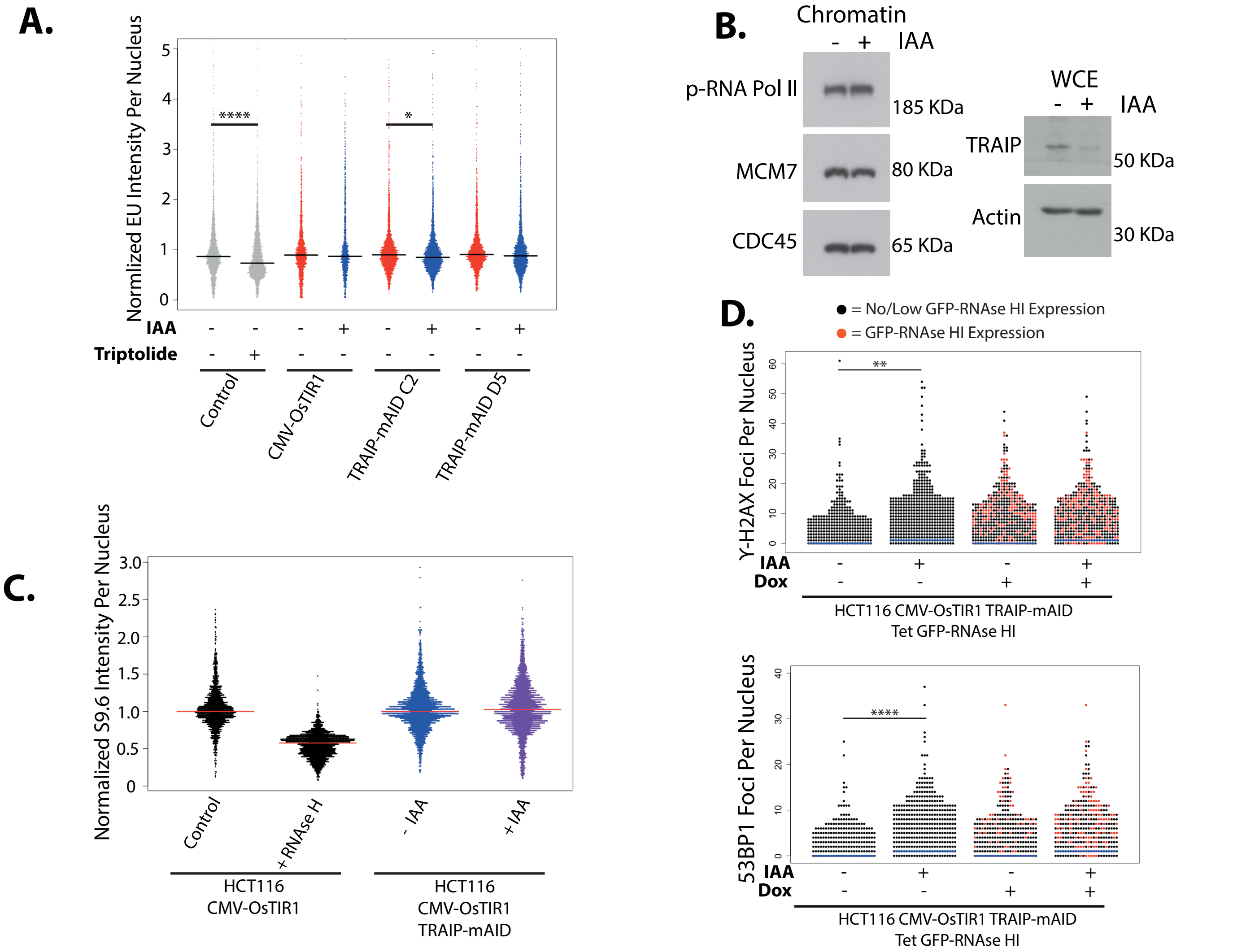

Supplementary Figure 11. Effects of TRAIP-mAID degradation on transcription. (A) Degradation of TRAIP-mAID does not increase global transcription levels. HCT116 TRAIP-mAID cells were arrested in G1, where TRAIP was degraded as described previously. Upon entry to S-phase (12 h post release), triptolide was optionally added to the control sample for 90 minutes. In all samples EU was added for 60 minutes 13.5 h following release from G1 arrest to label newly synthesised RNA. Incorporated EU was visualised using immunofluorescence following a copper-catalysed Click-IT reaction following manufacturers protocols. The median EU intensity per nucleus was quantified and normalised to the control (- IAA - Triptolide), shown as beeswarm plots. Statistical testing was conducted using pairwise t.tests: Control vs Triptolide treatment: $p < 0.0001$; TRAIP-mAID C2 - IAA vs + IAA: $p = 0.0102$. N = 3.

(B) No global increase of RNA Pol II on chromatin in absence of TRAIP. HCT116 TRAIP-mAID cells were arrested in G1, TRAIP optionally degraded and cells released into S-phase. In the middle of S-phase (12 h after G1 release) WCE and chromatin fractions were prepared and analysed by western blotting with indicated antibodies (n=3). **(C) No changes in R-loops level was detected upon TRAIP depletion.**

The S9.6 RNA:DNA hybrid antibody was used to detect any major changes to R-loop levels in the HCT116 degron cells. S-phase cells with and without TRAIP were stained for the S9.6 antibody and the respective fluorescence measured using Cell Profiler. Quantification of S9.6 intensity. **(D) Overexpression of RNAseH1 does not rescue DNA damage repair signals (γH2AX and 53BP1 foci) after TRAIP degradation.** HCT116 TRAIP-mAID cells were transduced with lentivirus to facilitate the Tet-inducible expression of GFP-RNAse HI. Asynchronous cells were treated optionally with DOX or IAA as indicated and fixed for DNA damage foci. Shown are the quantified number of foci per cell. Black circles are cells with no detectable GFP; cells with low or no GFP-RNAse HI expression. Red circles depict cells with detectable GFP; cells with GFP-RNAse HI expressed. Medians are shown by blue bar. Statistical analysis was carried out using t.tests. with significance shown on the plot (yH2AX - IAA vs + IAA: $p = 0.001709$; 3BP1 - IAA vs + IAA: $p = 0.0009167$. Source data are provided as a Source Data file.

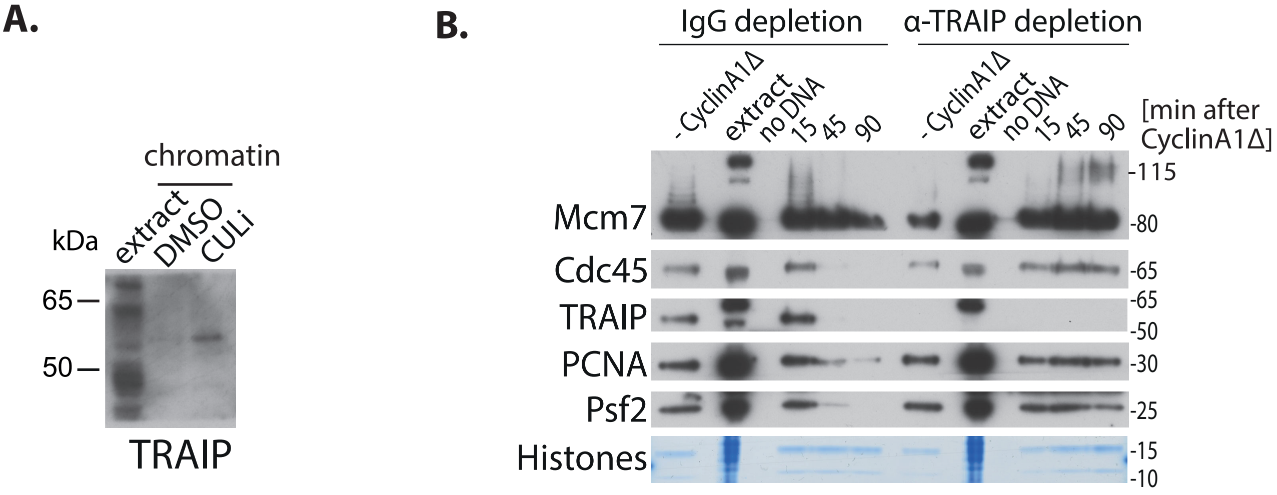

Supplementary Figure 12. TRAIP depleted Xenopus egg extract is unable to unload replisomes in mitosis. (A) Characterisation of *X.l* TRAIP antibody raised for this study. The level of TRAIP in the egg extract is low and all of our TRAIP antibodies recognise other bands in full egg extract. We have therefore also resolved samples of chromatin with accumulated replisomes (cullin inhibitor MLN4924 treatment for 90 min, CULi) to confirm that the band between 65 and 50 kD is indeed behaving as expected from TRAIP (n=3) **(B)** TRAIP depleted extract cannot unload replisomes in mitosis. Non-specific IgG or α-TRAIP depleted egg extract was used to replicate DNA to completion in presence of neddylation inhibitor MLN4924, which inhibits activity of Cullin type ubiquitin ligases and blocks S-phase pathway of replisome disassembly. Once replication was completed, extracts were supplemented with Cyclin A1Δ to stimulate progression into mitosis. Chromatin was isolated at indicated timepoints and replisome presence on chromatin was monitored by western blotting with indicated antibodies. In mock depleted extract CMGs (as visualised by Cdc45, Psf2) are removed from chromatin, while in α-TRAIP depleted egg extract they are retained on chromatin due to lack of TRAIP (n=4). Source data are provided as a Source Data file.

Supplementary Table 1. Summary of the significant GOTerms identified from the genes isolated using y-H2AX ChIP sequencing

Analysis Type	Gene Ontology Term	Count (# Genes)	Frequency (%)	Corrected P-Value (Benjamini)
Biological Process (GOTerm BP)	positive regulation of transcription from RNA polymerase II promoter	55	10.5	0.0014
Biological Process (GOTerm BP)	negative regulation of transcription from RNA polymerase II promoter	42	8	0.0077
Biological Process (GOTerm BP)	ephrin receptor signaling pathway	12	2.3	0.013
Biological Process (GOTerm BP)	vascular endothelial growth factor receptor signaling pathway	11	2.1	0.013
Biological Process (GOTerm BP)	transcription from RNA polymerase II promoter	31	5.9	0.034
Biological Process (GOTerm BP)	transcription, DNA-templated	81	15.4	0.045
Biological Process (GOTerm BP)	regulation of actin cytoskeleton organization	8	1.5	0.071
Biological Process (GOTerm BP)	covalent chromatin modification	12	2.3	0.071
Biological Process (GOTerm BP)	protein phosphorylation	27	5.1	0.08
Molecular Function (GOTerm MF)	protein binding	289	55	0.0026
Cellular Component (GOTerm CC)	nucleus	206	39.2	0.000000016
Cellular Component (GOTerm CC)	nucleoplasm	114	21.7	0.000047
Cellular Component (GOTerm CC)	cytoplasm	175	33.3	0.0041
Cellular Component (GOTerm CC)	cytosol	117	22.3	0.017
Biological Process (GOTerm BP)	positive regulation of transcription from RNA polymerase II promoter	55	10.5	0.0014
