# Supplementary material for: TRAIP resolves DNA replication-transcription conflicts during the S-phase of unperturbed cells
Source: Nat Commun. 2023 Aug 21;14:5071. doi: 10.1038/s41467-023-40695-y (PMC10442450; doi:10.1038/s41467-023-40695-y)
Supplement: Supplementary file 3 — Reporting Summary [file 41467_2023_40695_MOESM3_ESM.pdf]

nature portfolio

Corresponding author(s): Dr Aga Gambus

Last updated by author(s): Jul 24, 2023

Reporting Summary

Nature Portfolio wishes to improve the reproducibility of the work that we publish. This form provides structure for consistency and transparency in reporting. For further information on Nature Portfolio policies, see our Editorial Policies and the Editorial Policy Checklist.

Statistics

For all statistical analyses, confirm that the following items are present in the figure legend, table legend, main text, or Methods section.

n/a	Confirmed	
☐	☒	The exact sample size (*n*) for each experimental group/condition, given as a discrete number and unit of measurement
☐	☒	A statement on whether measurements were taken from distinct samples or whether the same sample was measured repeatedly
☐	☒	The statistical test(s) used AND whether they are one- or two-sided<br>*Only common tests should be described solely by name; describe more complex techniques in the Methods section.*
☒	☐	A description of all covariates tested
☐	☒	A description of any assumptions or corrections, such as tests of normality and adjustment for multiple comparisons
☐	☒	A full description of the statistical parameters including central tendency (e.g. means) or other basic estimates (e.g. regression coefficient) AND variation (e.g. standard deviation) or associated estimates of uncertainty (e.g. confidence intervals)
☒	☐	For null hypothesis testing, the test statistic (e.g. *F*, *t*, *r*) with confidence intervals, effect sizes, degrees of freedom and *P* value noted<br>*Give P values as exact values whenever suitable.*
☒	☐	For Bayesian analysis, information on the choice of priors and Markov chain Monte Carlo settings
☒	☐	For hierarchical and complex designs, identification of the appropriate level for tests and full reporting of outcomes
☒	☐	Estimates of effect sizes (e.g. Cohen's *d*, Pearson's *r*), indicating how they were calculated

Our web collection on statistics for biologists contains articles on many of the points above.

Software and code

Policy information about availability of computer code

Data collection	Flow Cytometry: Beckman CytExpert V. 2.5. Immunofluorescence: Leica LASX V. 3.7.4.23463, Real-time PCR: Thermo Fisher Connect
Data analysis	Flow Cytometry: FlowJo V. 10. Immunofluorescence: Leica LASX V. 3.7.4.23463, ImageJ V 2.1.0, CellProfiler V4.0.6. Statistics: RStudio V1.0.153, GraphPad Prism V9. Real-Time PCR: Microsoft Excel. ChIP-seq: Bowtie 2 v.2.4.2, MACS2 v.2.1.1, Galaxy, EaSeq

For manuscripts utilizing custom algorithms or software that are central to the research but not yet described in published literature, software must be made available to editors and reviewers. We strongly encourage code deposition in a community repository (e.g. GitHub). See the Nature Portfolio guidelines for submitting code & software for further information.

Data

Policy information about availability of data

All manuscripts must include a data availability statement. This statement should provide the following information, where applicable:

- Accession codes, unique identifiers, or web links for publicly available datasets
- A description of any restrictions on data availability
- For clinical datasets or third party data, please ensure that the statement adheres to our policy

Source data are provided with this paper.
The accession number for all genomic data files reported in this paper is GEO: GSE201158
https://www.ncbi.nlm.nih.gov/geo/query/acc.cgi?acc=GSE201158

Field-specific reporting

Please select the one below that is the best fit for your research. If you are not sure, read the appropriate sections before making your selection.

- [x] Life sciences
- [ ] Behavioural & social sciences
- [ ] Ecological, evolutionary & environmental sciences

For a reference copy of the document with all sections, see nature.com/documents/nr-reporting-summary-flat.pdf

Life sciences study design

All studies must disclose on these points even when the disclosure is negative.

Sample size	As a standard practice for the field, statistical analysis were performed on data derived from at least 3 biological replicates.
Data exclusions	no data are excluded from the analysis
Replication	data is derived from at least 3 biological repeats, all repeats were successful and where possible data used to perform statistical analysis
Randomization	samples were allocated to groups depending on cell treatments as described in the manuscript.
Blinding	where possible samples were scored blind.

Reporting for specific materials, systems and methods

We require information from authors about some types of materials, experimental systems and methods used in many studies. Here, indicate whether each material, system or method listed is relevant to your study. If you are not sure if a list item applies to your research, read the appropriate section before selecting a response.

Materials & experimental systems

n/a	Involved in the study	
☐	☒	Antibodies
☐	☒	Eukaryotic cell lines
☒	☐	Palaeontology and archaeology
☐	☒	Animals and other organisms
☒	☐	Human research participants
☒	☐	Clinical data
☒	☐	Dual use research of concern

Methods

n/a	Involved in the study	
☐	☒	ChIP-seq
☐	☒	Flow cytometry
☒	☐	MRI-based neuroimaging

Antibodies

Antibodies used

Antibodies for Immunofluorescence:
Mouse anti-Ser139 γ-H2AX (Sigma-Aldrich, JBW301; 1:1000)
Rabbit anti-53BP1 (Novus Biologicals NB100-904; 1:1000)
Mouse anti-Mitosin (BD Biosciences 610768; 1:300)
Rabbit anti-P-Histone H3S10 (Cell Signalling 9701; 1:1000)
AF488 anti-mouse secondary (Invitrogen A32723; 1:1000)
AF555 anti-rabbit secondary (Invitrogen A21428; 1:1000)
Rabbit anti-S9.6 (Francis Crick Institute; 1:200)

Antibodies used for Flow Cytometry:
Rabbit anti-P-Histone H3S10 (Cell Signalling 9701; 1:500)
Mouse anti-MCM7 (Santa Cruz 9966; 1:500)
Mouse anti-BrdU (BD Biosciences 347580, clone B44; 1:5)
AF488 anti-mouse secondary (Invitrogen A32723; 1:1000)
AF555 anti-rabbit secondary (Invitrogen A21428; 1:1000)
AttoN647 anti-rabbit secondary (Sigma-Aldrich 40839; 1:500)

Antibodies used for ChIP-Seq:
1 μg of rabbit anti-Ser139 γ-H2AX (Abcam 29893)
1 μg of rabbit anti-H2AX (Merck Millipore 07627)

Antibodies used for Immunoblotting:
Anti-TRAIP antibodies were kindly provided by Prof. N. Mailand and used 1:300 in 5% Milk in TBST.

Mouse anti-β Actin loading controls (Santa Cruz sc-47778, C4, HRP-conjugated; 1:5000 in 5% BSA in TBST).
Rabbit anti-UBXN7 (Thermo Fisher Scientific 15779771; 1:1000) in 5% Milk in TBST.
Rabbit Anti-SPRTN (Novus Biologicals NBP1-84163; 1:1000) in 5% Milk in TBST.

Antibodies used for PLA assays:
Anti-elongating RNA Polymerase II (serine 5): Mouse anti-Rpb1 CTD (Cell Signalling 2629S, 4H8; 1:250)
Rabbit anti-biotin (Bethyl Laboratories A150-109A; 1:500)
Rabbit anti-GFP (Chromotek PABG1; 1:250)
Rabbit anti-AND1 (Novus Biological NBP1-89091; 1:250)
Mouse anti-GFP (Roche 11814460001; 1:250)

Antibodies used for Xenopus work:
Mouse anti-PCNA (Sigma P8825; 1:2000)
Rabbit anti-TRAIP (Novus Biologicals NBP1-87125; 1:500)
Rabbit anti-P-Chk1 (S345) (Cell Signalling 2341; 1:1000)
Rabbit anti-Ser139 γ-H2AX (Trevigen 4418-APC-020; 1:1000)
Affinity purified anti-Cdc45, anti-Psf2 63, anti-Mcm7 21 and anti-GINS antibody 64 were previously described.
Affinity purified anti-TRAIP is described in Supplementary Figure 12.

Validation	The validation of antibodies raised against Xenopus laevis proteins in-house have been described previously in Xenopus egg extract system, and as such are referenced appropriately in the text. Human TRAIP antibodies, were validated in previous manuscripts as cited. all other commercially available antibodies were validated for the assays we used them for and the validation is provided online on the manufactures' websites.

Eukaryotic cell lines

Policy information about cell lines

Cell line source(s)	All cell lines used were originally sourced from Prof. M. Kanemaki's lab, as detailed and referenced in the text: Natsume, T., Kiyomitsu, T., Saga, Y. & Kanemaki, M. T. Rapid Protein Depletion in Human Cells by Auxin-Inducible Degron Tagging with Short Homology Donors. Cell reports 15, 210-218 (2016). https://doi.org:10.1016/j.celrep.2016.03.001
Authentication	For derivatives of the original cells generated during this study the genotype was verified by PCR amplification and sequencing of the relevant loci and the protein levels established by western blotting with the appropriate antibodies.
Mycoplasma contamination	All cell lines were tested for Mycoplasma using the EZ-PCR Mycoplasma testing kit (Biological Industries). Negative results were confirmed by including a +(ve) control provided by the kit.
Commonly misidentified lines (See ICLAC register)	No commonly misidentified lines were used in this study.

Animals and other organisms

Policy information about studies involving animals; ARRIVE guidelines recommended for reporting animal research

Laboratory animals	Xenopus laevis
Wild animals	*Provide details on animals observed in or captured in the field; report species, sex and age where possible. Describe how animals were caught and transported and what happened to captive animals after the study (if killed, explain why and describe method; if released, say where and when) OR state that the study did not involve wild animals.*
Field-collected samples	*For laboratory work with field-collected samples, describe all relevant parameters such as housing, maintenance, temperature, photoperiod and end-of-experiment protocol OR state that the study did not involve samples collected from the field.*
Ethics oversight	home office project and personal license<br>University of Birmingham Ethics committee

Note that full information on the approval of the study protocol must also be provided in the manuscript.

ChIP-seq

Data deposition

☒ Confirm that both raw and final processed data have been deposited in a public database such as GEO.

☒ Confirm that you have deposited or provided access to graph files (e.g. BED files) for the called peaks.

Data access links<br>*May remain private before publication.*	https://www.ncbi.nlm.nih.gov/geo/query/acc.cgi?acc=GSE201158
Files in database submission	D H2AX unind

D H2AX unind-2
D gH2AX unind
D gH2AX unind-2
D H2AX +IAA
D H2AX +IAA-2
D gH2AX +IAA
D gH2AX +IAA-2

Genome browser session (e.g. UCSC)

no longer applicable

Methodology

Replicates

2 replicates for each condition uninduced and following addition of IAA

Sequencing depth

For all files, 76 bases SE reads.
D H2AX unind: Total 29091109, mapped 23811723.
D H2AX unind-2: Total: 17820390, mapped 15211091
D gH2AX unind: Total: 43121600, mapped 41383012.
D gH2AX unind-2: Total: 20561203, mapped 19488354.
D H2AX +IAA: Total 45128111, mapped 41393047.
D H2AX +IAA-2: Total : 20885308, mapped 17527330.
D gH2AX +IAA: 55773966, mapped, mapped 54011294.
D gH2AX +IAA-2: Total: 21102714, mapped 19631808.

Antibodies

Rabbit polyclonal anti-histone H2AX, Merck Millipore, Cat# 07627
Rabbit polyclonal anti-Phospho-Histone H2A.X (Ser139), Abcam, Cat#ab2893

Peak calling parameters

-m 8 30, -p 0.00001

Data quality

Quality of the sequencing was assessed by FASTQC. Peaks were identified according to the parameters above; only peaks identified in both the experimental repeats were further characterized into details.

Software

EaSeq.net

Flow Cytometry

Plots

Confirm that:

- [x] The axis labels state the marker and fluorochrome used (e.g. CD4-FITC).
- [x] The axis scales are clearly visible. Include numbers along axes only for bottom left plot of group (a 'group' is an analysis of identical markers).
- [x] All plots are contour plots with outliers or pseudocolor plots.
- [x] A numerical value for number of cells or percentage (with statistics) is provided.

Methodology

Sample preparation

For un-extracted cells, following the required experimental procedures (e.g., proliferation curves) cells were harvested and fixed in 70% ethanol in PBS for 16 hours at – 20C. Following fixation, cells were washed twice in washing buffer (5% BSA, 0.1% Tween-20, PBS) and antibody staining carried out if required. Briefly, washed cells were resuspended in 100l primary antibody in washing buffer and incubated at room temperature for 1 hour, rocking to prevent cells from settling. The cells were washed twice in washing buffer and re-suspended in 100l secondary antibody in washing buffer for 1 hour at room temperature in the dark. Stained cells were washed 1X in washing buffer and 2X in D-PBS (-) before being resuspended in either Hoechst Staining Buffer (5g/ml Hoechst 33582, PBS) or Propidium Iodide Staining Buffer (50g/ml Propidium Iodide, 50g/ml RNase A, PBS). For BrdU detection, 10M of BrdU was added to the growth media 1 hour prior to harvesting. Cells were collected and fixed in ethanol as described. Fixed cells were washed once in PBS before being resuspended in 1 ml 2 M HCL supplemented with 0.1 mg/ml Pepsin for 20 minutes. Cells were then washed, and antibody staining carried out as described. To explore the replisome binding pattern on chromatin, cells were extracted using CSK buffer (25 mM HEPES pH 7.4, 50 mM NaCl, 3 mM MgCl2, 300 mM Sucrose, 0.5% Triton X-100, 1X complete protease inhibitors) to remove soluble fractions. The protocol used to extract cells has been described elsewhere (Forment & Jackson, 2015).

Instrument

All flow cytometry was carried out using a Beckman CytoFlex Instrument.

Software

Software used: Beckman CytExpert V. 2.5. and FLowJo V.10

Cell population abundance

N/A as we were not isolating specific cells within a population.

Gating strategy	Gating strategies were as detailed in the paper. Briefly: Control cells were gated to isolate the main cell population using FSC-A vs SSC-A. This population was then further gated to remove doublets twice (First using FSC-A vs FSC-H and then DNA content stain - A vs DNA content stain - H). Finally the particular region of interest (+ve) signal was gated using unstained controls. All gates applied using a control sample were then set to all samples.

[x] Tick this box to confirm that a figure exemplifying the gating strategy is provided in the Supplementary Information.
